# Activities of fungal metabolites sphaeropsidin A and B to fight against canine coronavirus

**DOI:** 10.1007/s11259-026-11377-z

**Published:** 2026-06-26

**Authors:** Luca Del Sorbo, Maria Michela Salvatore, Valentina Iovane, Rosa Giugliano, Okri Fréjus Hans Ohouko, Anna Andolfi, Alessio Buonavoglia, Filomena Fiorito

**Affiliations:** 1 Department of Veterinary Medicine and Animal Production, University of Naples Federico II, Naples, 80137 Italy; 2 Department of Agricultural Sciences, University of Naples Federico II, Portici, 80055 Italy; 3 Department of Experimental Medicine, University of Campania L. Vanvitelli, Naples, 80138 Italy; 4 Research Unit in Applied Microbiology and Pharmacology of Natural Substances, Laboratory of Research in Applied Biology, University of Abomey-Calavi, Cotonou, Benin; 5 Department of Chemical Science, University of Naples Federico II, Naples, Italy; 6 Department of Human Sciences, LINK Campus University, Rome, 00165 Italy

**Keywords:** CCoV, Sphaeropsidins, AhR, A72, Antiviral activity, In vitro

## Abstract

Pimarane diterpenes represent a notable class of metabolites. Secondary metabolites sphaeropsidin A (SphA) and sphaeropsidin B (SphB), produced by phytopathogenic fungi, have been recently reported for their potential efficacy against bovine coronavirus (BCoV). Hence, for discovering natural antivirals to serve as alternatives to conventional drugs, in this study the efficacy of SphA and SphB during infection with canine coronavirus (CCoV-II) in A72 cell line was examined. Our results demonstrated that a significant reduction in virus yield and in gene and protein expression of viral nucleocapsid protein (NP) was detected in sphaeropsidins (Sphs)-treated infected cells. In infected groups both Sphs enhanced cell viability and improved cellular morphology as well as cytoskeleton rearrangements. Moreover, the protein expression of aryl hydrocarbon receptor (AhR), a strategic modulator of CoVs infection, and of its target CYP1A1, was markedly downregulated in the presence of SphA and SphB during infection. These results were accompanied by a beneficial deacidification of lysosomal environment in infected cells treated with SphA and SphB compared to untreated-infected groups. Overall, our findings demonstrated a promising action of SphA and SphB towards CCoV infection in vitro.

## Introduction

### Overview of canine coronavirus

Among coronaviruses, the alphacoronavirus canine coronavirus (CCoV) has been causing significant concern in both dogs and humans. CCoV is an enteric dog pathogen, also known as canine enteric coronavirus (CECoV), that discriminates it from canine respiratory coronavirus (CRCoV) (Olarte-castillo et al. 2025). CCoV comprises two genotypes, namely canine coronavirus type I (CCoV-I) and canine coronavirus type II (CCoV-II). Analyzing amino acid sequence in the N-terminal region of spike (S) protein, emerged that CCoV-I is genetically related to feline coronavirus type I (FCoV-I), while genetic and phylogenetic analysis revealed that CCoV-II and transmissible gastroenteritis virus (TGEV) originated from a common ancestor (Pratelli et al. 2020, 2021; Odigie et al. 2024; Olarte-castillo et al. 2025). In dogs, CCoVs infection typically cause high morbidity but low mortality. They are mainly spread through the faecal–oral route and provoke severe gastroenteritis, diarrhoea, and vomiting, especially in puppies. However, in some cases, CCoVs leads to fatal disease due to co-infections with other pathogens, such as canine parvovirus type 2 (CPV-2), canine adenovirus type 1 or canine distemper virus (Patelli et al. 2001; Decaro et al. 2004; Buonavoglia et al. 2023). Particularly, cases of CCoV-associated mortality in the absence of CPV infection have been also detected in puppies (Evermann et al. 2005). Furthermore, a hypervirulent recombinant strain of CCoV-II, called pantropic strain, originating from canine-feline-porcine sources (CB/05), was reported in Italy in 2005. It has been associated with multisystemic infections characterized by severe lesions across multiple organs, leading to systemic disease and fatal outcomes in pups (Buonavoglia et al. 2006; Timurkan et al. 2021). Then, pantropic strain has been also isolated in dogs from other European or extra-European countries like Brazil (Decaro et al. 2013; Pinto et al. 2014; Alfano et al. 2020).

The ability of CoVs to infect a wide range of host cells is a key factor in tissue tropism. Due to its tendency to mutate and to make recombination, CCoV can lead to the emergence of new genetic strains, some of which show high pathogenicity and capability for jumping species barriers. Intriguingly, the recent emergence of a new FCoV strain, named FCoV-23, which provoked the recent outbreak of feline infectious peritonitis in Cyprus, is due to a recombination between a FCoV-1 strain with the pantropic CCoV-2, NA/09 (Attipa et al. 2025). In humans, a new strain named CCoV-HuPn-2018 was identified in a child hospitalized with pneumonia in Malaysia (Vlasova et al. 2022). Interestingly, the viral genome exhibited approximately 97% nucleotide sequence identity of its structural genes when compared with CCoV-II. However, analysis of S gene revealed the presence of genomic regions derived from feline coronavirus (FCoV) and TGEV, supporting the hypothesis of a complex recombination event underlying its origin (Gray et al. 2026). Moreover, the same strain of CCoV-HuPn-2018 was also isolated from nasopharyngeal swabs of 18 among 200 people presenting pneumonia symptoms in Vietnam from January 2020 to July 2022 (Phan et al. 2025; Gray et al. 2026).

As above, the ability of CoVs to mutate and recombine their genomes across species barriers has been extensively demonstrated (Theamboonlers et al. 2007; Lednicky et al. 2021; Vlasova et al. 2022; Phan et al. 2025; Gray et al. 2026). As the frequency of viral mutations makes therapeutic interventions ineffective, the development of targeted therapies remains a priority for global public health. The emergence of hypervirulent strains is driving the development of new potential drugs targeting new pathways (Kim et al. 2025; Vicente et al. 2025). Natural products, particularly those from fungi, including fungal secondary metabolites (SMs), have been attracting increasing interest due to their diverse properties. Indeed, they are valuable therapeutic molecules that possess antibacterial, immunomodulatory, antifungal, and antiviral activities, potentially overcoming existing treatment challenges (Wellensiek et al. 2013; Linnakoski et al. 2018). In this context, numerous studies reported the potential antiviral activity of SMs useful to fight against human and animal coronaviruses (Pang et al. 2021; Cerracchio et al. 2022a, 2023, 2024; Fiorito et al. 2022; Hamed et al. 2024; Del Sorbo et al. 2025d).

### Aryl hydrocarbon receptor in CoVs infections

About new mechanisms of action of potential antivirals, it has been recently reported that CoVs, during infection, provoke an activation of aryl hydrocarbon receptor (AhR) (Grunewald et al. 2020; Liu et al. 2020; Giovannoni et al. 2021; Cerracchio et al. 2022b; Shi et al. 2023; Yousefi et al. 2023; Wang et al. 2024; Del Sorbo et al. 2025a; Sorbo et al. 2025c; Zhang et al. 2025). AhR is a transcription factor expressed by different cell types. Its activation following binding with exogenous or endogenous ligands, including environmental pollutants (Hu et al. 2023b; Barreira-Silva et al. 2024; Del Sorbo et al. 2025b; Grycová et al. 2025; Sahoo et al. 2025), leads to AhR translocation by translocator ARNT from the cytoplasm to the nucleus, where it plays an important role in gene regulation by activating genes, such as cytochrome P450 1A1/cytochrome P450 1B1 (CYP1A1/CYP1B1), involved in immunomodulation pathway (Hu et al. 2023b; Barreira-Silva et al. 2024; Grycová et al. 2025; Sahoo et al. 2025). This process leads to cytokine secretion and modulation of immune responses (Hu et al. 2023b; Barreira-Silva et al. 2024; Grycová et al. 2025). Also during viral infections, AhR interacts with viral proteins, regulating the immune system (Giovannoni et al. 2021; Hu et al. 2023b; Barreira-Silva et al. 2024; Grycová et al. 2025). Indeed, an activation of AhR signaling has been also observed in infections by multiple human and animal RNA virus such as influenza A virus and Zika virus besides CoVs (Liu et al. 2020; Zeng et al. 2020; Giovannoni et al. 2021; Cerracchio et al. 2022b; Hu et al. 2023a; Barreira-Silva et al. 2024; Del Sorbo et al. 2025a; Sorbo et al. 2025c; Grycová et al. 2025). Interestingly, anti-CoVs activity due to the treatment with some natural SMs also provoked an AhR modulation (Cerracchio et al. 2022a, 2023, 2024; Fiorito et al. 2022; Del Sorbo et al. 2025d).

### Endo-lysosomal pathway in CoVs infections

It is well established that enveloped viruses, including several coronaviruses, exploit the endo-lysosomal pathway to enter host cells. Recent evidence demonstrates that CoVs infection in Vero cells is associated with a significant downregulation of genes encoding lysosomal membrane proteins, along with those implicated in the regulation and maintenance of lysosomal acidification (Gassen et al. 2019). Furthermore, CoVs infection has been associated with compromised lysosomal function, as evidenced by the decrease in lysosomal acidification in infected Vero, MDBK, CRFK, and A72 cell lines (Gassen et al. 2019; Ghosh et al. 2020; Cerracchio et al. 2023; Del Sorbo et al. 2025d; Sorbo et al. 2025c). Interestingly, due to the treatment with various natural and synthetic substances, a further deacidification of lysosome was appreciated during CoVs infection (Cerracchio et al. 2023; Del Sorbo et al. 2025d; Sorbo et al. 2025c). Therefore, the search for agents that can modulate this pathway may represent an important pharmacological target.

### Biological activities of fungal metabolites sphaeropsidin A and sphaeropsidin B

**Fig. 1:**
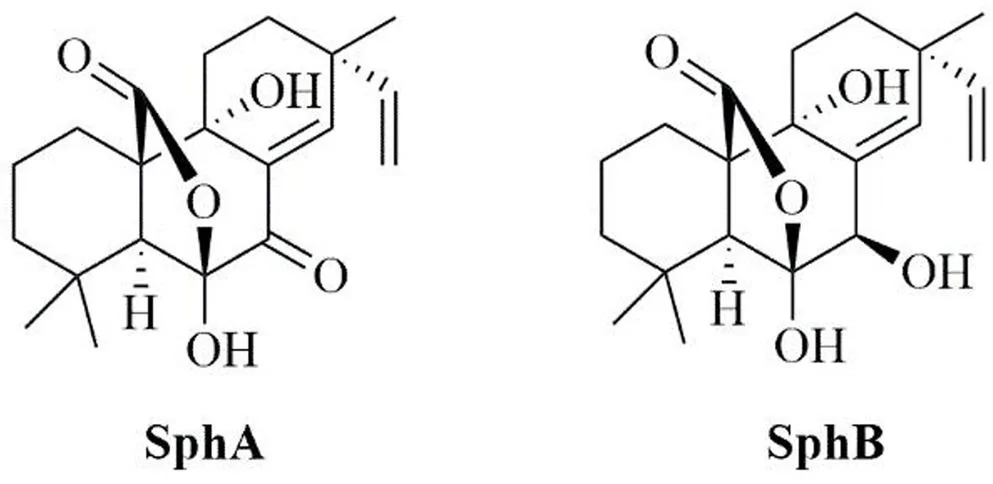
Chemical structures of SphA and SphB

Here, we investigated the impact of the fungal metabolites sphaeropsidin A (SphA) and sphaeropsidin B (SphB) (Fig. 1) during CCoV infection. SphA and SphB are pimarane diterpene, produced by various fungal pathogens belonging to the *Botryosphaeriaceae* family (Salvatore et al. 2023) and known for their antifungal (Buonanno et al. 2024), insecticidal (Di Lelio et al. 2022), anticancer (Wagh et al. 2025), antibiofilm (Roscetto et al. 2020) and antiviral (Khan et al. 2021; Del Sorbo et al. 2025d) properties. Specifically, antiviral activities of SphA and SphB against BCoV have been investigated in in vitro and in silico studies (Del Sorbo et al. 2025d). As only very few reports have evaluated the role of SphA/B as potential antivirals compounds, for overcoming this research gap in literature, we explored the action of SphA and SphB in CCoV infection, also highlighting the innovative aspects of this study.

## Materials and methods

### Isolation and identification of SphA and SphB

SphA and SphB were isolated from cultures of the fungus *Diplodia corticola* (MAEC10) and identified via nuclear magnetic resonance (NMR) spectroscopy as previously reported (Salvatore et al. 2023).

### Cell cultures and virus infection

A72 cells cultures were carried out in Dulbecco’s modified Eagle’s minimal essential medium (DMEM) with supplement of 10% foetal bovine serum (FBS) and incubated at 37 °C and 5% CO_2_ (Cerracchio et al. 2024). CCoV type II (strain S/378, GenBank accession number KC175341CCoV), was cultured and titrated in A72 cells.

SphA and SphB were solubilized in DMSO (Sigma-Aldrich, St. Louis, MI, USA) to have a dose of 5000 µM (stock solution). Then, concentrations of 0.5, 1, 2.5, 5, 10 and 50 µM of SphA and SphB in DMEM were prepared in order to obtain the final doses added to culture cells. DMSO in DMEM (0.1% *v/v*) was used as vehicle control.

Monolayers of A72 cells were infected or not with CCoV strain S/378 with a titer of 1 × 10^6.92^/mL tissue culture of infectious doses 50 (TCID_50_), at a multiplicity of infection (MOI) of 0.5 and treated with DMEM supplemented with 10% FBS containing different concentrations of both SphA and SphB (0.5, 1, 2.5, 5, 10 and 50 µM) to have six groups: (a) untreated uninfected cells; (b) untreated infected cells; (c) SphA-treated uninfected cells; (d) SphB-treated uninfected cells; (e) SphA-treated infected cells; (f) SphB-treated infected cells. After 1 h of CCoV adsorption at 37 °C, cells were incubated and processed at 24 h post infection (p.i.). CCoV remained in the culture medium throughout the experiment. CCoV actively replicates in A72 cells within 24–48 h (Cerracchio et al. 2024; Del Sorbo et al. 2025b), reaching 90% of the title already after 24 h (personal observations). However, as reported in the Funding paragraph, this work was supported by two different Research Projects, in which we reported investigating some mechanisms of action at intermediate times.

### Cell viability

Cell viability was performed on A72 cells treated with different concentrations of SphA and SphB for 24 h through MTT test (Cell Proliferation Kit I; Roche, Basel, Switzerland) (Abolhassani et al. 2014; Fiorito et al. 2022; Del Sorbo et al. 2025a). A72 cells were plated in 96-well plates at a density of 5 × 10³ cells per well and incubated at 37 °C. After 24 h, cell monolayers were exposed to various increasing concentrations of SphA and SphB (0.5, 1, 2.5, 5, 10, and 50 µM). After 24 h, 10 µL of MTT solution (5 mg/mL) was added to each well according to the manufacturer’s instructions. Absorbance was recorded at 540–560 nm using a microplate reader (Thermo Scientific™ Multiskan™ FC, Thermo Fisher Scientific, Waltham, MA, USA).

Cell viability (IC_50_) calculations for the A72 cells, as well as the concentration of the effective 50% (CC_50_) values for SphA and SphB, which correspond to a 50% inhibition of the viral replication, were evaluated using the IC_50_ Calculator provided by AAT Bioquest (https://www.aatbio.com/tools/ic50-calculator, accessed on 14 July 2025). The mean logarithmic IC_50_ value was derived as the logarithm of the highest tested concentration minus one-third of the difference between the logarithms of the maximum and minimum concentrations applied (Sebaugh 2011). Cell viability was expressed as the percentage of viable cells over the total number of cells, according to the following formula:

$$\:cell\:viability=\frac{absorbance\:of\:treated\:cells}{absorbance\:of\:untreated\:cells\:}\times\:100$$

and data were reported as mean ± standard deviation from three independent experiments.

### Viral inhibition assay

Cell viability during viral infection was assessed using the MTT assay. A72 cells were infected with CCoV at a MOI of 0.5 and simultaneously treated or not with SphA and SphB at different not cytotoxic concentrations (0.5, 1, and 2.5 µM). At 24 h post-infection (p. i.), cell viability was determined as described above.

### Examination of cell morphology and cell cytoskeleton

To assess cell morphology, A72 cells were treated or not with SphA and SphB at concentrations of 2,5 µM and 1 µM, respectively, and infected or not with CCoV (MOI 0.5). After 24 h of infection, cells were processed for acridine orange/propidium iodide (AO/PI) staining (Giugliano et al. 2025a), as well as for and Phalloidin staining (Wulf et al. 1979; Del Sorbo et al. 2026). To verify that the virus acted on the cytoskeleton of CCoV-infected cells, we carried out Phalloidin staining in the presence of a specific viral marker anti-NP monoclonal mouse, MAB 938 (The Native Antigen Company, Kidlington, UK) (1:200) and goat anti-mouse Alexa Fluor 594 (Thermo Fisher Scientific) (1:500).

### Immunofluorescence staining

A72 cells were treated or not with SphA (2.5 µM) as well as SphB (1 µM) and infected or not with CCoV at MOI of 0.5. At 24 h p.i., immunofluorescence staining for AhR, CYP1A1, and NP were assessed as reported (Del Sorbo et al. 2025d). Images were captured with the ZOE Fluorescent Cell Imager (Bio-Rad Laboratories) and analysed with the ImageJ software (National Institutes of Health), as reported (Del Sorbo et al. 2026).

### Virus production

A72 cells were seeded in a 24-well plate, treated or not with SphA (2.5 µM) and SphB (1 µM), and infected or not with CCoV at an MOI of 0.5. After 24 h, three cycles of freezing and thawing were performed, and the cells were collected and stored at −80 °C. Virus titer was determined by the TCID_50_ method, according to Reed and Muench (1938), as previously reported (Nastri et al. 2024; Giugliano et al. 2025b). In brief, 10-fold dilutions of the cell lysates in complete DMEM were used to infect a confluent monolayer of A72 cells, with four replicates for each dilution. Serial dilutions of CCoV were added to A72 cells cultured in 96-well plates, and after 24 h, the cells were monitored for CPE by using an inverted optical microscope. Four wells in each plate served as virus-free controls. The titer was then calculated based on the number of wells showing CPE after incubation at 37 °C for 24 h, using the Reed-Muench method (Nastri et al. 2024; Giugliano et al. 2025b). In addition, CPE was assessed at 24 h p.i., after methanol fixation and crystal violet staining (0.1% w/v) (Sigma-Aldrich, St. Louis, MI, USA) (Giugliano et al. 2025a).

### Real-time-PCR

A72 cells, were treated or not with SphA and SphB, and were infected with CCoV (MOI 0.5) for 24 h. To assess the expression levels of the gene encoding the viral NP, A72 cells, treated or not with SphA and SphB, were infected with CCoV (MOI 0.5) for 24 h. To assess the expression levels of the gene encoding the viral NP, total RNA was collected, retrotranscribed into cDNA and Real-time PCR was performed, as previously described (Giugliano et al. 2025a). The Relative Ct (the threshold cycle) of the gene of interest was normalized to the housekeeping gene (GAPDH). The following primers were used for Real-time PCR: for NP gene, forward primer TTGATCGTTTTTATAACGGTTCTACAA and reverse primer AATGGGCCATAATAGCCACATAAT; for GAPDH gene, forward primer CCTTTCATTGAGCTCCAT and reverse primer CGTACATGGGAGCGTC. Finally, the mRNA levels were calculated using the 2 − ΔΔCt method.

### LysoRed staining

CCoV infected cells at MOI of 0.5, treated with SphA (2.5 µM), and SphB (1 µM), and incubated for 24 h, were stained with CytoPainter LysoRed Indicator Reagent (Abcam Cambridge, UK), and incubated, following the user manual. After that, the cells were washed and analysed by microscopic ZOE Fluorescent Cell Imager (Bio-Rad Laboratories, Hercules, CA, USA) (Del Sorbo et al. 2026).

### Statistical analysis

Data normality was assessed before One-way ANOVA with Tukey’s post-test and Student’s t test, which were assessed by using GraphPad Prism version 10.0 (GraphPad Software Inc., San Diego, CA). *P* < 0.05 was statistically significant. Results are expressed as mean ± S.D.

## Results

### SphA and SphB increased cell viability during CCoV infection

**Fig. 2:**
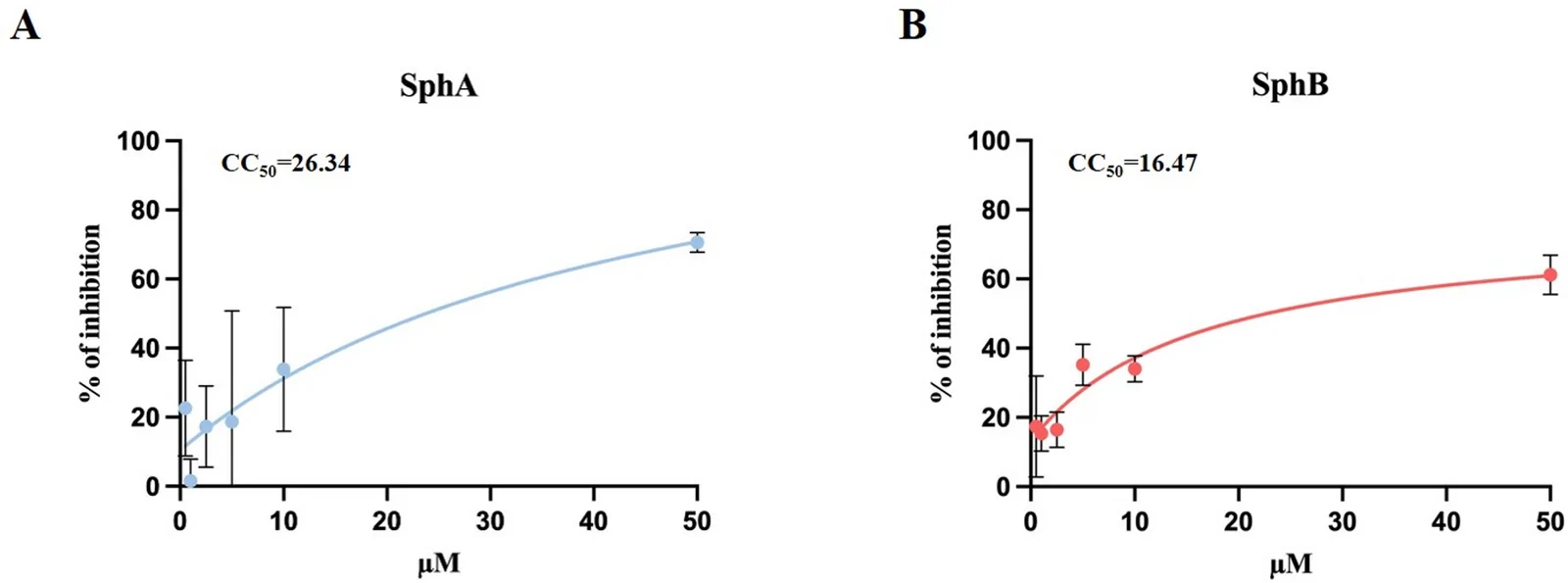
Evaluation of cell viability by MTT assay of Sphs after 24 h of treatment on the A72 cell line. The CC_50_ values of SphA (**A**) and SphB (**B**) were determined by exposing A72 cells to a range of concentrations (0.5, 1, 2.5, 5, 10 and 50 µM). Dose–response curves were generated after 24 h of incubation. For both compounds, cell viability was evaluated using MTT test. The results, expressed as a percentage of inhibition, were reported as the mean ± S.D. of triplicate measurements. Inhibition rates were compared to the DMSO-treated control and analyzed using GraphPad Prism 10.5.0 through non-linear regression with a dose–response inhibition model (Three parameters). Each point on the curves represents the mean ± S.D. of three replicates

For cell viability, the MTT assay was performed on the A72 cell line using different doses of SphA and SphB (0.5, 1, 2.5, 5, 10, and 50 µM). After 24 h of treatment, a dose–response curves were generated using the cytotoxic concentrations of these molecules that resulted in a 50% reduction in A72 cell viability (CC₅₀) (Fig. 2). Cell viability was expressed as a percentage relative to the control, resulting in CC_50_ values of 26.34 µM for SphA and 16.47 µM for SphB (Fig. 2).

**Fig. 3:**
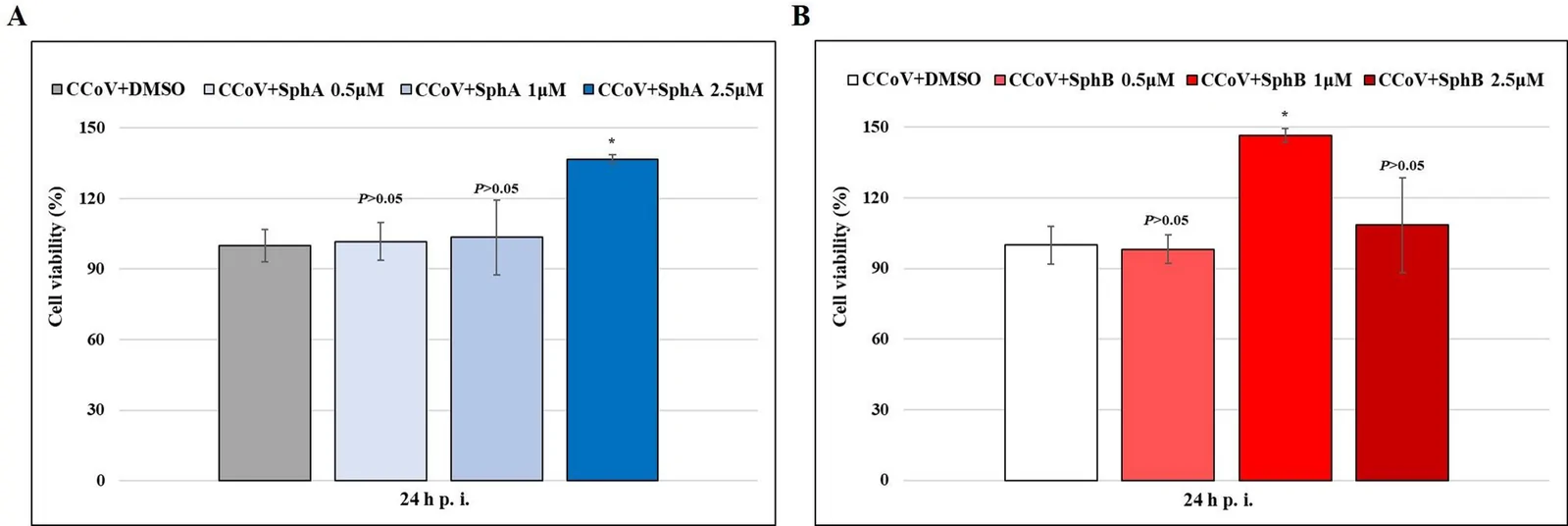
Following CCoV infection, cell viability was enhanced by SphA (**A**) and SphB (**B**). After 24 h of infection A72 infected cells treated with SphA and SphB (0.5, 1 and 2.5 µM), were processed by MTT test. Bars illustrating the dose–response effects of the concentrations of SphA and SphB on A72 cells infected with CCoV. Viable cell number was expressed as a percentage of the total cells, and data are presented as the mean ± S.D. of triplicate experiments. Statistical analysis was performed using one-way ANOVA followed by Tukey’s post hoc test. Significant differences between CCoV + DMSO and either CCoV + SphA or CCoV + SphB treatment groups are highlighted; probabilities: *, *P* < 0.05

Subsequently, A72 cells were infected with CCoV at MOI of 0.5 and then treated or not with the non-cytotoxic concentrations of both metabolites (0.5, 1 and 2.5 µM). In Fig. 3, we present and evaluate the effects of concentrations of SphA and SphB (0.5, 1 and 2.5 µM) on CCoV-infected cells. Following 24 h of treatment with SphA at 2.5 µM and SphB at 1 µM resulted in a significant increase (*p* < 0.05) in A72 cell viability compared to infected controls (Fig. 3).

The effects of selected concentrations of SphA and SphB on CCoV-infected cells (Fig. 3) indicated that SphA at 2.5 µM (Fig. 3A) and SphB at 1 µM (Fig. 3B) significantly increased (*P* < 0.05) cell viability in A72 infected cells after 24 h of treatment, compared to infected controls (Fig. 3).

### SphA and SphB reduced morphological marks of cell death during CCoV infection in A72 cells

**Fig. 4:**
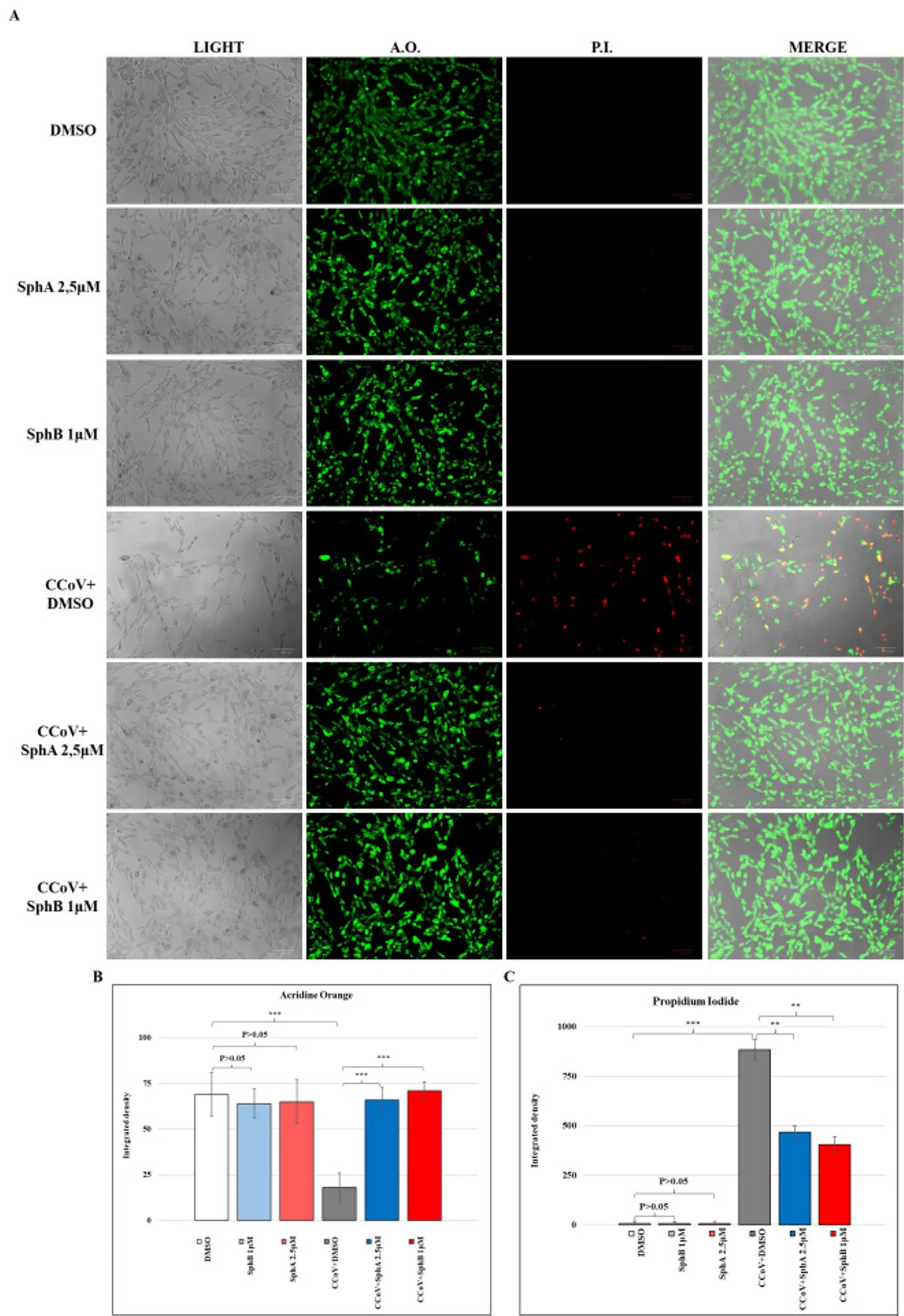

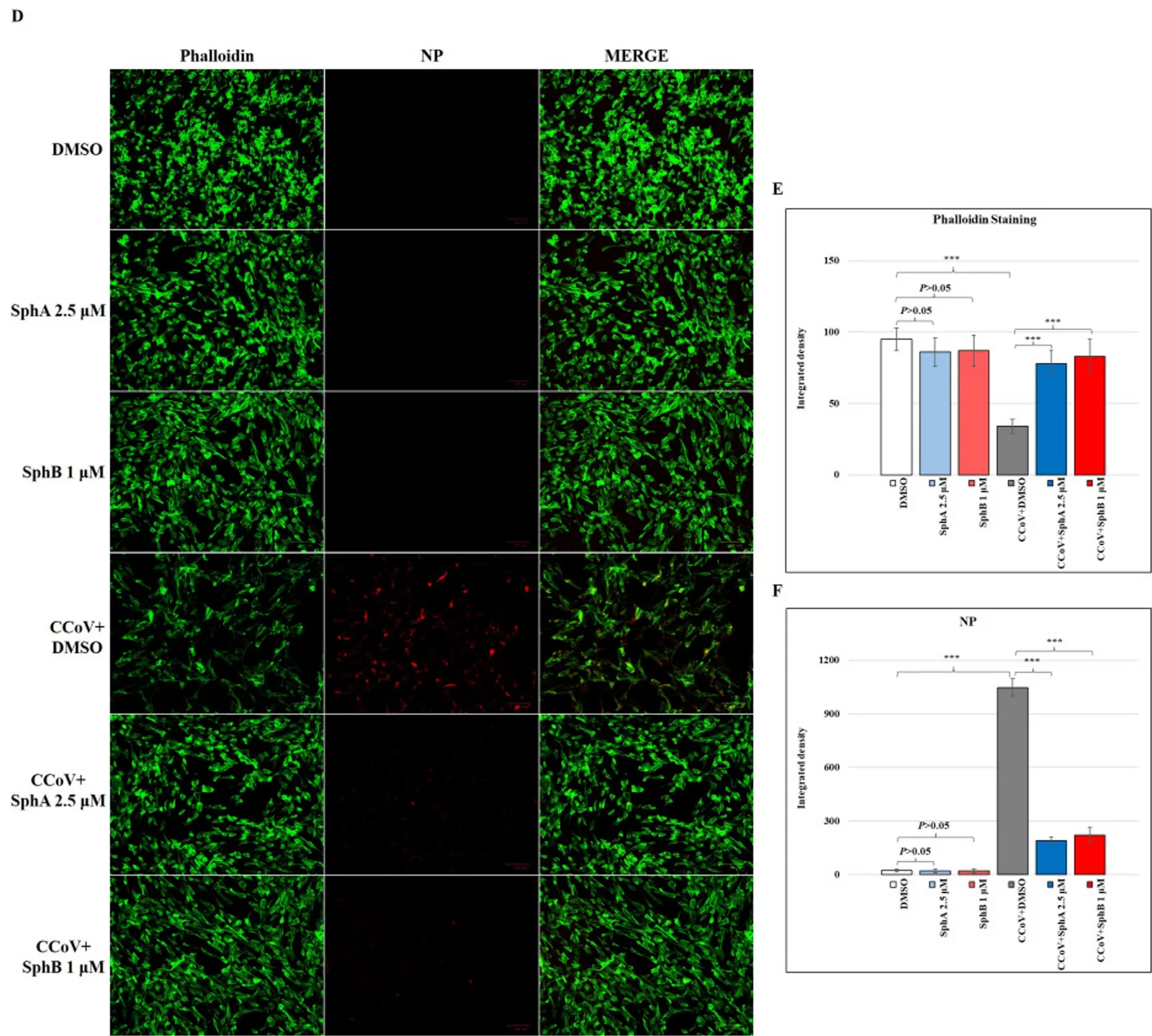
SphA and SphB reduced the morphological marks of cell death and promoted cytoskeletal organization in A72 cells during CCoV infection. Cells were treated or untreated with SphA and SphB, then infected with CCoV (MOI = 0.5) and further incubated with or without SphA (2.5 µM) and SphB (1 µM) for 24 h. **A**) In AO/PI staining panels, PI-positive fluorescent cells, indicative of dead or dying cells, were primarily detected in the CCoV-infected group, whereas their number was markedly reduced in the infected group treated with SphA and SphB. **B**-**C**) Fluorescence intensity analysis revealed that, compared with the CCoV + DMSO group, green fluorescence was significantly higher and red fluorescence markedly lower in the CCoV + SphA and CCoV+SphB groups. No evidence of cell death was found in the DMSO or SphA and SphB groups. D-F) Phalloidin-stained actin filaments of cell cytoskeleton (green), and anti-Canine Coronavirus nucleocapsid antibody-stained N protein (red). Phalloidin-stained samples showed comparable cytoskeletal structures in the DMSO, SphA and SphB groups. A pronounced decrease in fluorescence intensity was detected in the CCoV-infected cells, whereas treatment with SphA and SphB restored cytoskeletal organization, as evidenced by the enhanced green, fluorescent signal in infected cells. In these groups a significant reduction of fluorescent red (NP) signal was detected. Significant differences between the CCoV-infected group and the SphA- or SphB-treated groups are indicated by probability *P*. ***, *P* < 0.001). Fluorescence density was quantified from representative images (scale bars: 25 μm and 100 μm). Results are shown from one of three independent experiments, *n* = 3

In the AO/PI panels, viable cells, stained green were predominantly observed in the CCoV + SphA and CCoV + SphB groups, with levels similar to the control. Fewer viable cells were detected in the CCoV + DMSO group (Fig. 4A-B). PI-positive fluorescent cells, indicating dead or dying cells, were mainly found in CCoV-infected samples, but their numbers decreased significantly in CCoV-infected cells treated with SphA and SphB (Fig. 4A-C). Actin filaments of cell cytoskeleton were stained by Phalloidin in green, and NP viral protein was stained in red (4D). In CCoV-infected cells stained with Phalloidin, a notable rearrangement of the cytoskeleton was observed, characterized by a significant decrease in fluorescence density (Fig. 4D-E). The presence of both Sphs enhanced the organization of actin in the cytoskeleton of these infected cells, which were characterized by an evident increase in green fluorescence intensity (Fig. 4D-E), as well as by a substantial reduction in red (NP) signal (Fig. 4D-F).

These results indicated that SphA and SphB protect A72 cells following CCoV infection.

### SphA and SphB reduced virus yield in A72 cells during CCoV infection

**Fig. 5:**
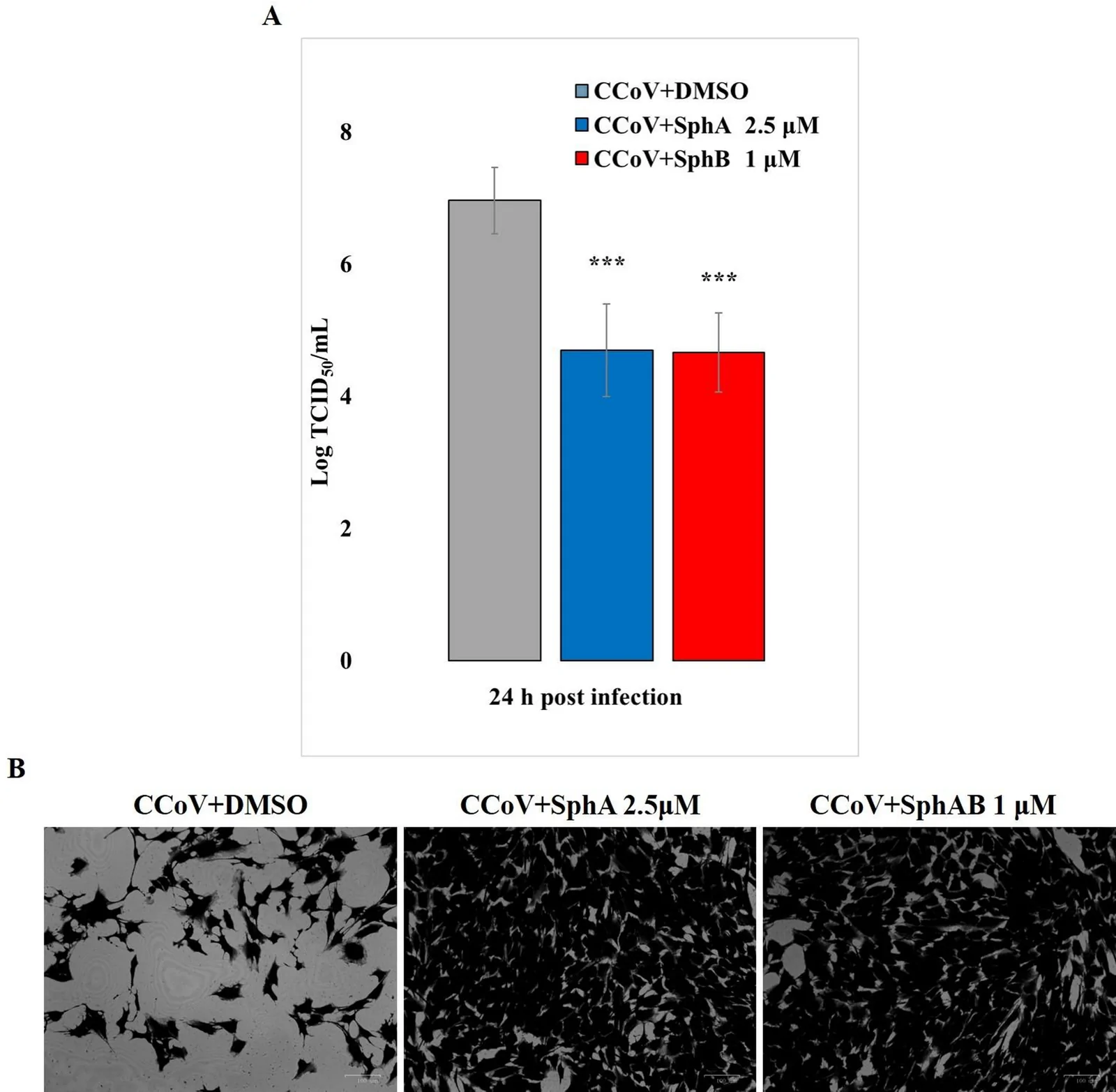
SphA and SphB reduced viral yield during CCoV infection in A72 cells. A72 cells were infected with CCoV and treated or untreated with SphA (2.5 µM) and SphB (1 µM) for 24 h p.i. (**A**) Viral production was quantified using the TCID₅₀ assay and expressed as Log TCID₅₀/mL. Statistical analysis was performed using Student’s t-test. Significant differences between the CCoV-infected group and the SphA- or SphB-treated groups are indicated (***, *P* < 0.001). (**B**) Cytopathic effects (CPE) were evaluated by crystal violet staining and visualized with a ZOE Cell Imager. Scale bar represents 100 μm. The data shown represent one experiment representative of three independent replicates

During CCoV infection in A72 cells, treatment with SphA (2.5 µM) and SphB (1 µM) significantly influenced viral production. At 24 h post-infection, CCoV titer (expressed as logarithmic values) was markedly reduced (*P* < 0.001) in cells treated with SphA and SphB compared to the CCoV + DMSO control group (Fig. 5A).

Microscopic examination of the A72 infected cells, after performing crystal violet staining showed a pronounced cytopathic effect (CPE) in untreated cells compared with the SphA- and SphB-treated groups (Fig. 5B). Overall, these results demonstrate that both fungal metabolites, SphA and SphB, significantly reduced CCoV yield during infection in A72 cells.

### SphA and SphB reduced NP gene and protein expression during CCoVinfection in A72 cells

**Fig. 6:**
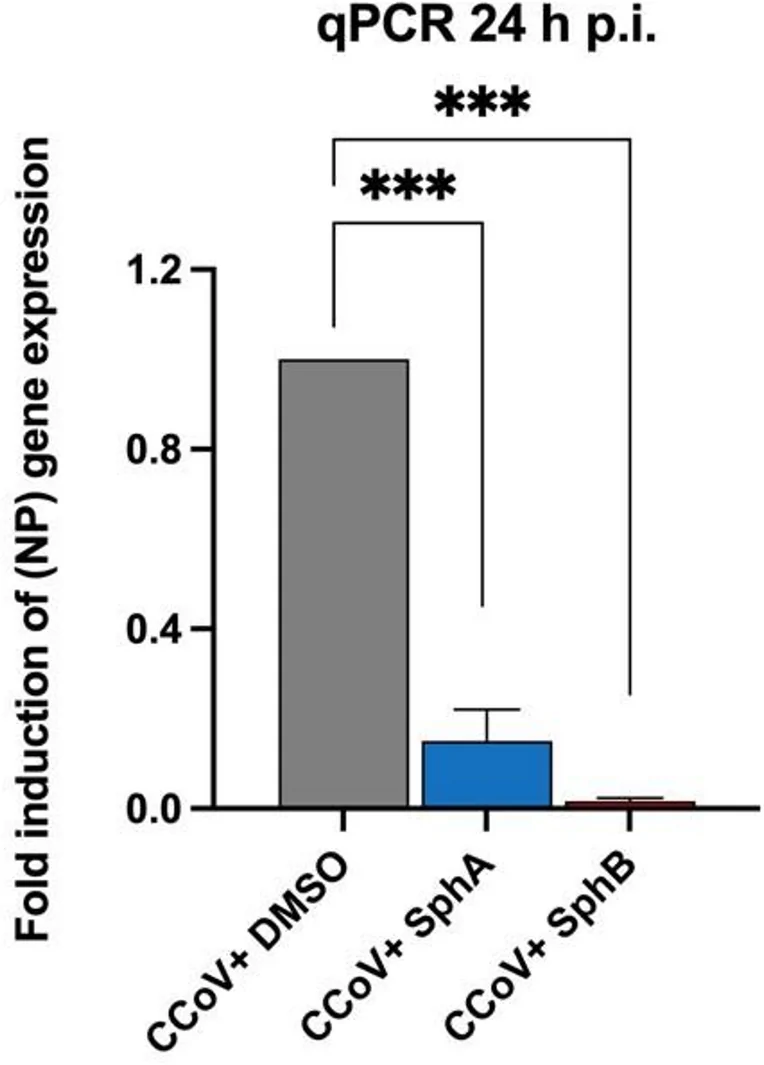
SphA and SphB reduced NP gene expression during CCoV infection in A72 cells. qPCR analysis of NP transcripts following treatment with SphA and SphB showed that both metabolites significantly decreased NP gene expression compared with untreated CCoV-infected cells. Error bars indicate the standard deviations of the measurements. Statistical analyses were determined by ordinary one-way ANOVA with Dunnett’s test for multiple comparisons. Significant differences in NP expression between the CCoV + DMSO group and the CCoV + SphA or CCoV + SphB groups are marked *** *P* < 0.001. The data presented correspond to one experiment representative of three independent replicates

Real-Time analysis revealed that SphA and SphB treatments significantly reduced the relative expression of viral nucleoprotein (NP) compared to untreated CCoV-infected cells. Both metabolites exhibited a clear antiviral effect at the gene level, with SphB showing a stronger inhibitory activity than SphA (Fig. 6).

**Fig. 7:**
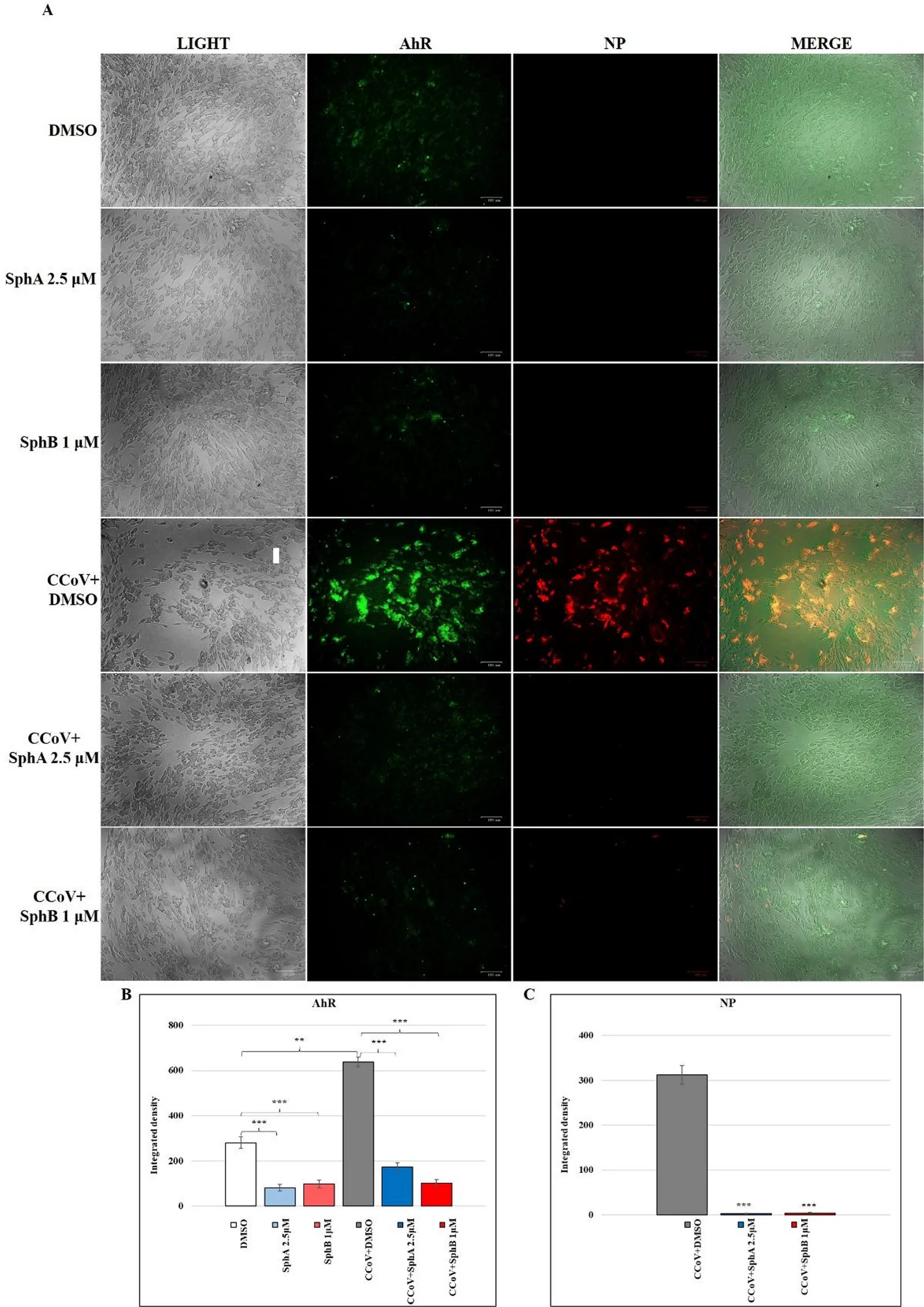
SphA and SphB downregulated the expression of AhR and NP proteins during CCoV infection in A72 cells. A72 cells were infected with CCoV at an MOI of 0.5 and either treated or left untreated with SphA (2.5 µM) and SphB (1 µM) for 24 h. (**A**) AhR and NP protein levels were evaluated in both treated and untreated CCoV-infected groups using immunofluorescence staining. Scale bar: 100 μm. (**B**, **C**) Bar graphs display the mean ratios derived from the integrated densities of AhR and NP protein expression during CCoV infection. Statistical analyses were performed using one-way ANOVA followed by Tukey’s post hoc test. Significant differences between DMSO-treated controls and CCoV-infected cells, as well as between CCoV-infected cells and the SphA- or SphB-treated infected groups, are indicated (*** *P* < 0.001; ** *P* < 0.01). Integrated densities were quantified using ImageJ (National Institutes of Health; Java 1.8.0_345). Error bars represent standard deviations. Results are shown from one of three independent experiments, *n* = 3

NP protein expression was also assessed during CCoV infection in A72 cells (Fig. 7). NP levels were markedly reduced in infected groups treated with either SphA or SphB compared with untreated infected cells (Fig. 7A). This observation was further confirmed by the quantification of integrated fluorescence density (Fig. 7C), which showed a significant decrease (*P* < 0.001).

### SphA and SphB induced downregulation in AhR signaling during CCoV infection

CCoV infection leads to the activation of AhR (Fig. 7A) as previously reported (Cerracchio et al. 2024). Whereas, during CCoV infection, immunofluorescence staining showed that AhR expression was reduced in A72 cells treated with SphA (2.5 µM) and SphB (1 µM) (Fig. 7A). Consequently, the integrated fluorescence densities for AhR were significantly decreased (*P* < 0.05), as illustrated in Fig. 7B.

**Fig. 8:**
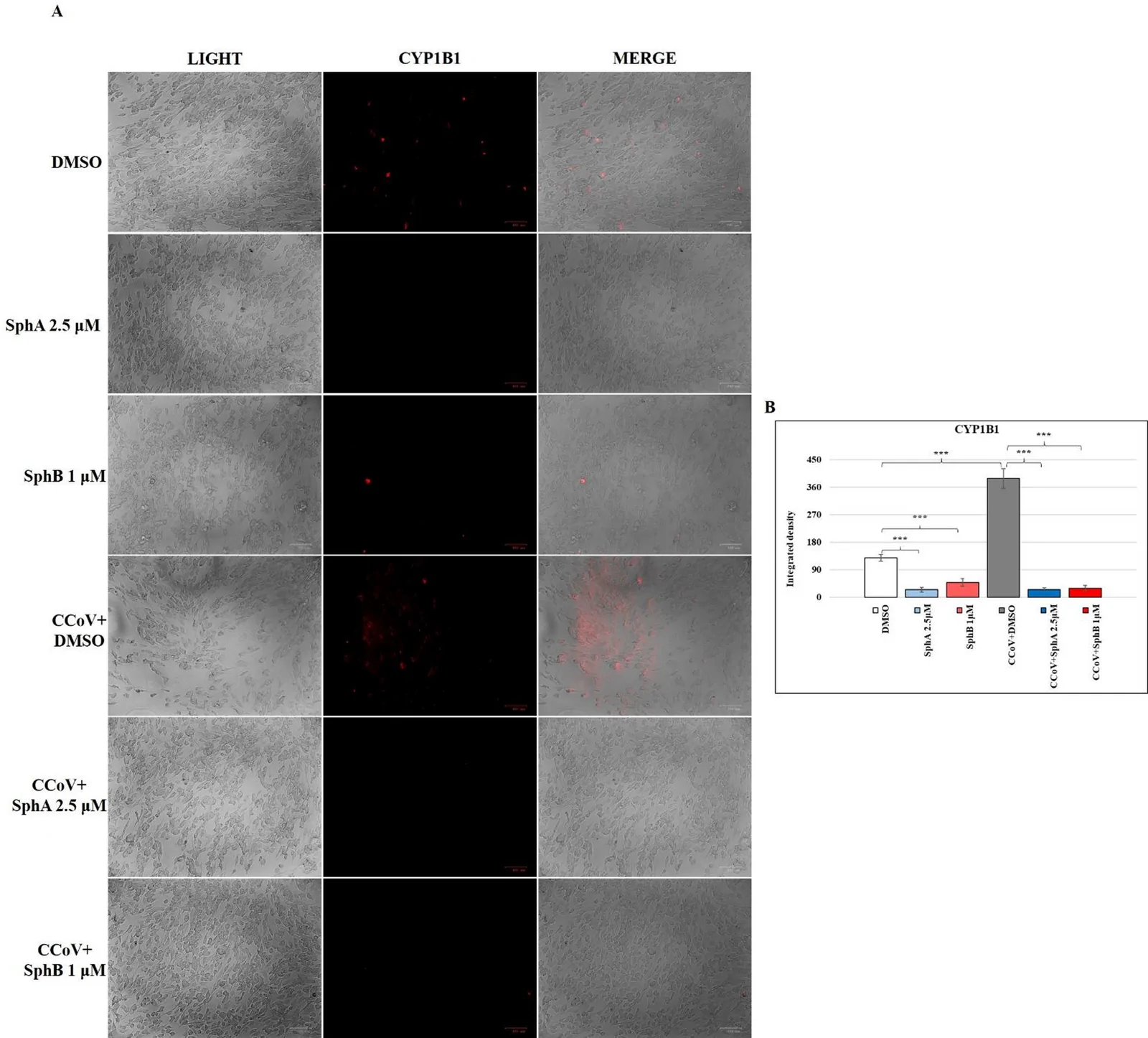
SphA and SphB downregulated CYP1B1 expression (AhR signaling) during CCoV infection in A72 cells. A72 cells were infected with CCoV at an MOI of 0.5 and treated with SphA and SphB for 24 h. (**A**) Immunofluorescence staining was performed using antibodies against CYP1B1. Scale bar: 50 μm. (**B**) Bar graphs display the mean ratios derived from the integrated densities of CYP1B1 expression during CCoV infection in the presence of SphA and SphB. Statistical analysis was conducted using one-way ANOVA followed by Tukey’s post hoc test. Significant differences between DMSO and CCoV-infected cells, as well as between CCoV-infected cells and the SphA- or SphB-treated groups, are indicated (*** *P* < 0.001). Integrated densities were quantified using ImageJ. Error bars represent standard deviations. Results are shown from one of three independent experiments, *n* =3

To assess the effects of SphA and SphB on AhR signaling, the expression of the Cytochrome CYP1B1 was also examined during CCoV infection in A72 cells. The enhancement of CYP1B1 induced by CCoV was significantly diminished following treatment with SphA and SphB (Fig. 8A). This reduction was further confirmed by the integrated fluorescence density analysis, which showed a significant decrease in CYP1B1 levels in response to both metabolites (*P* < 0.001), as illustrated in Fig. 8B.

### SphA and SphB induced lysosomes deacidification during CCoV infection in A72 cells

**Fig. 9:**
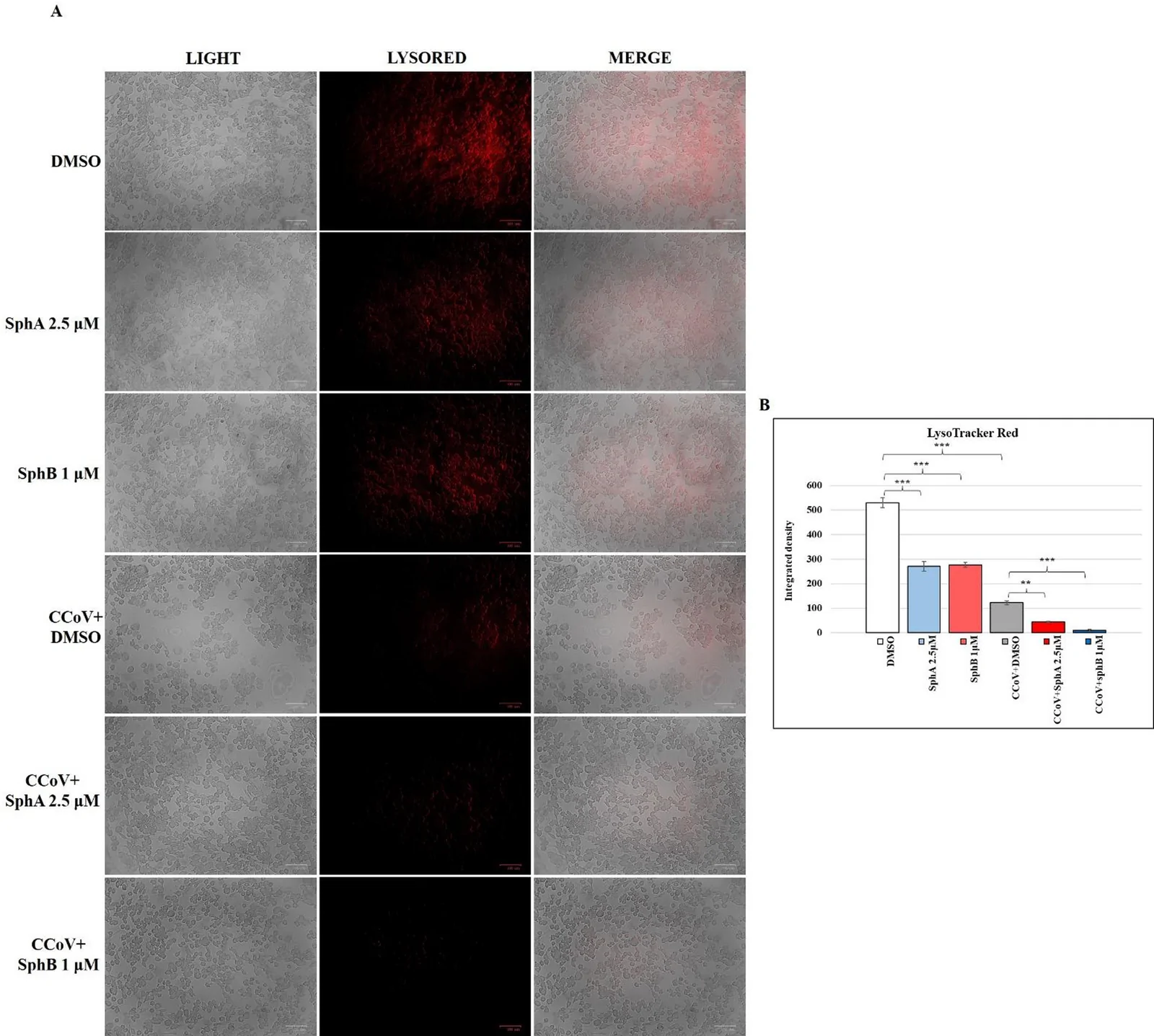
SphA and SphB induce lysosomal deacidification during CCoV infection in A72 cells. A72 cells were infected with CCoV at an MOI of 0.5 and treated with SphA (2.5 µM) or SphB (1 µM) for 24 h. (**A**) LysoRed staining shows DMSO controls, untreated CCoV-infected cells, and CCoV-infected cells treated with SphA or SphB. Scale bar: 100 μm. (**B**) Quantification of lysosomal acidity was performed using integrated LysoRed densities in ImageJ. Bars represent mean ratios ± S.D. from triplicate measurements. One-way ANOVA with Tukey’s post hoc test revealed significant differences between DMSO controls and SphA or SphB groups (*** *P* < 0.001), DMSO controls and CCoV-infected cells (*** *P* < 0.001), as well as between CCoV-infected cells and both SphA- and SphB- infected-treated groups (** *P* < 0.01, *** *P* < 0.001). Results are shown from one of three independent experiments, *n* = 3

The acidic environment of lysosomal organelles was assessed during CCoV infection using LysoRed staining, a marker for identifying lysosomes in live cells. We observed in DMSO-treated control cells an acidic lysosomal environment was observed, which was reduced following CCoV infection (Fig. 9A, B). Treatment of CCoV-infected cells with SphA (2.5 µM) or SphB (1 µM) induced an additional decrease in lysosomal acidity (Fig. 9A, B).

## Discussion

In this study, two secondary metabolites, SphA and SphB, isolated from *D. corticola*, were evaluated during infection with CCoV type II, CCoV-S/378 strain, in A72 cells, a suitable cell line for exploring CCoV in in vitro infection (Regan et al. 2020). Although we had previously tested the antiviral activity of SphA and SphB in BCoV (Del Sorbo et al. 2025d), sometimes, some potentially antiviral substances are active against some CoVs but not against other ones. Hence, we suppose that a simple translational advantage over our previous work could be not sufficient, but experimental tests were required to verify the efficacy of SphA and SphB against CCoV. As above reported, CCoV is an α-CoV and is closely related to FCoV and TGEV (Pratelli et al. 2020, 2021; Odigie et al. 2024; Olarte-castillo et al. 2025). Because it is an α-CoV, it is only distantly related to BCoV, a β-CoV (Everest et al. 2022). In addition, it has been recently demonstrated that using 3’,4’-dimethoxy-α-naphthoflavone (DiMNF), a small molecule inhibitor of AhR, the compound selectively inhibits HCoV-OC43 (β-CoV) infection but not SARS CoV-2 (β-CoV) (Yousefi et al. 2023).

Following CCoV infection for 24 h, the co-treatment with non-toxic doses of 2.5 µM for SphA and 1 µM for SphB, increased the viability of infected groups in A72 cells, which also resulted in improved morphological features following AO/PI staining. Fluorescent Phalloidin was used to detect morphological modifications on actin filaments, one of the cytoskeletal polymers that constitute cytoskeleton. The structural and dynamic properties of the cytoskeleton enable cells to execute a broad spectrum of integrated functions, including mediating interactions with the extracellular environment, coordinating force generation for cell motility and morphological remodeling, facilitating vesicular trafficking throughout the cytoplasm, and ensuring the spatial organization and compartmentalization of intracellular components (Wickstead and Gull 2011; Wen et al. 2020). Herein, CCoV infection caused morphological modifications of actin in A72 cells. Interestingly, previous studies have shown that other alphacoronaviruses, such as TGEV and porcine hemagglutinating encephalomyelitis virus (PHEV), at the early stage of infection, could cause F-actin polymerization and rearrangement, which promote virus entry (Hu et al. 2016; Lv et al. 2019). In this study, the treatment with both Sphs positively improved the organization of actin in the cytoskeleton of CCoV infected cells. Therefore, the protective effect due to Sphs also demonstrated their role in contributing to the maintenance of cytoskeleton structure in infected cells. Similar protective effects were also provoked by 6-pentyl-α-pyrone, another fungal metabolite obtained by *Trichoderma atroviride*, in bovine cells (MDBK) during BCoV infection (Del Sorbo et al. 2026).

The treatment with SphA and SphB caused a substantial decrease in viral yield, accompanied by the reduction in gene and protein expression of the viral protein NP. A similar trend, with promising results, was previously reported in a research, demonstrating an antiviral action of SphA and SphB during BCoV infection in MDBK cells (Del Sorbo et al. 2025d).

Here, during CCoV infection, an activation of AhR signaling was observed. This mechanism of action also confirmed previous results showing the upregulation of AhR during CCoV infection (Cerracchio et al. 2022b). As above, AhR pathway is commonly activated by CoVs infection, such as α-CoVs, like human coronavirus (HCoV)−229E, FCoV and Porcine Epidemic Diarrhea Virus (PEDV) (Giovannoni et al. 2021; Wang et al. 2024; Del Sorbo et al. 2025c), β -CoVs, including SARS-CoV-1, SARS-CoV-2, mouse hepatitis virus (MHV), HCoV-OC43, Middle East respiratory syndrome (MERS)-CoV, and BCoV (Grunewald et al. 2020; Giovannoni et al. 2021; Shi et al. 2023; Yousefi et al. 2023; Del Sorbo et al. 2025a) as well as γ -CoVs like avian infectious bronchitis virus (IBV) (Zhang et al. 2025). In the presence of SphA and SphB, a downregulation in the levels of both AhR as well as its target protein CYP1B1 was found in infected groups. A similar trend, with a protective effect, was induced by a specific AhR inhibitor, CH223191, during CCoV infection in A72 cells (Cerracchio et al. 2022b). Based on the AhR signaling modulation, these results also reinforced the promising antiviral properties of SphA and SphB, fungal SMs also useful to counteract BCoV infection in vitro (Del Sorbo et al. 2025d). Remarkably, it has been recently demonstrated that CCoV replication is also related to formyl peptide receptor 2 (FPR2), which is another modulator of immune response (Giugliano et al. 2025a). Hence, FPR2, that results controlled by both host cell and by virus, may be proposed as a suitable target to further study the antiviral action of SphA and SphB to fight CCoV infection.

CoVs infect host cells by fusing with plasma membranes or endo-lysosomal membranes. Once inside endosomes, they depend on a carefully regulated acidic environment to activate proteases. These proteases cleave and induce maturation of structural viral proteins, so facilitating fusion with the endosomal membrane. This process contributes to the release of the viral genome from nucleocapsid into the cytoplasm, as well as to the replication (Mao et al. 2022). Overall, these mechanisms suggest that the endosomal pathway could be a promising drug target against CoVs infections. Here, during CCoV infection, we observed lysosomal deacidification in A72 cells, which were further deacidified following SphA and SphB treatments. Interestingly, comparable effects were observed in MDBK cells infected with BCoV and treated with SphA and SphB (Del Sorbo et al. 2025d). The activity of SphA and SphB on lysosomes was not easy to understand, but future studies could further clarify this action.

This work only focused on the virus which was not removed after the adsorption period and remained in the culture medium throughout the incubation. Consequently, it is difficult to distinguish whether the observed effects are due to the inhibition of intracellular viral replication or merely due to the direct inactivation/virucidal effect of the compounds on the free viral particles in the supernatant. Other limitations of this study, such as the absence of animal experiments and the lack of mutagenesis strain tests. However, these points could be the object of future work.

In conclusion, our work demonstrates that SphA and SphB could represent new antiviral agents against CCoV infection. Moreover, the modulation of AhR signaling, induced by those fungal metabolites, provides an interesting target for counteract CoVs. In this perspective, further studies, as in silico approach and investigation of alternative pathways are needed to elucidate the molecular mechanisms by which SphA and SphB interact with the canine AhR receptor.

## Data Availability

No datasets were generated or analysed during the current study.
